# The human microbiome in the 21^st^ century

**DOI:** 10.1038/s41467-020-18983-8

**Published:** 2020-10-16

**Authors:** Elze Rackaityte, Susan V. Lynch

**Affiliations:** 1 Department of Biochemistry and Biophysics, University of California, San Francisco, San Francisco, CA USA; 2 Division of Gastroenterology, Department of Medicine, University of California, San Francisco, San Francisco, CA USA

**Keywords:** Biotechnology, Microbiome

## Abstract

The human body supports a thriving diversity of microbes which comprise a dynamic, ancillary, functional system that synergistically develops in lock-step with physiological development of its host. The human microbiome field has transitioned from cataloging this rich diversity to dissecting molecular mechanisms by which microbiomes influence human health. Early life microbiome development trains immune function. Thus, vertically, horizontally, and environmentally acquired microbes and their metabolites have the potential to shape developmental trajectories with life-long implications for health.

Our recent understanding that the human body supports a thriving diversity of microbial life has led to a greater appreciation of the expanded functional gene capacity of the human superorganism. Over the past several years, the field of human microbiome research has transitioned from cataloging the diversity of these microorganisms to the view that they comprise a dynamic ancillary functional system that synergistically develops across spatial and temporal gradients in parallel with physiological development and decline^1–5^. For centuries, we have observed how human health is influenced by microbes and that interactions between microbial and host cells govern infectious diseases. What is becoming more apparent is that a wide array of conditions ranging from chronic inflammatory^6,7^ and metabolic^8–10^ diseases to neurological disorders^11,12^ and cancer^13^ have now been associated with microbiome functional perturbations. These may occur either locally at the site of disease manifestation or at remote mucosal sites or organ systems, which stimulate metabolic and immunologic changes in the host.

## Insights from germ-free mice

In 1885 Louis Pasteur speculated that gnotobiotic or germ-free (GF) animals would not be able to survive due to the extensive co-evolutionary history with microbes^14^. While GF animals can indeed be reared, studies using such animals have been instrumental in illustrating the crucial role of microbes in mammalian development and health. GF mice exhibit shorter lifespans, heavily enlarged caeca^15^, lack natural antibodies^16,17^, and are severely deficient in vitamin K and B12^15^. GF mice generated in genetically susceptible backgrounds enable investigation of relationships between host genetics and the microbiome. For example, conventional IL-10 deficient mice develop spontaneous colitis, but their GF counterparts neither develop colitis nor immune system activation^18^ pointing to the microbiome as a disease trigger in a system poised for inflammation. However, conventionalization of GF mice does not always rescue the observed defects. Upon experimental conventionalization in adults, previously GF mice display increased susceptibility to pathogens^19^. Supporting these findings, GF mice exhibit aberrant invariant natural killer T-cell function and increased morbidity in models of IBD and allergic asthma, which can only be rescued by conventionalization in the neonatal period but not in adulthood^20^. Thus, age-sensitive microbial programming appears critical to the establishment and training of immune function and subsequent health outcomes. Evidence that microbiomes and their products are responsible for human disease has also been provided using GF mice, in which transfer of patient microbiomes confers features of the disease phenotype on the recipient animal^21–25^.

## Microbial metabolites influence physiology

A large number of extrinsic and intrinsic factors, including diet, antimicrobials and immunity influence human microbiomes, in particular the gut microbiome which houses the largest number and diversity of microbes. In turn, the bioactive products of the microbiome shape human cell function locally^26,27^ and at remote sites. In studies of mono-colonized GF mice, members of the intestinal microbiome were shown to strongly influence mammalian energy harvest and metabolism^28,29^ and produce a suite of microbial-specific metabolites in physiologically relevant concentrations, a large number of which enter the circulation^30^. Thus, microbial activities at one body habitat may influence physiological conditions and cell function at a remote site. For example, increased concentrations of trimethylamine N-oxide (TMAO) associate with arthrosclerosis and is dependent on gut bacterial metabolism of dietary phosphatiylcholine^31,32^, providing the first evidence for a diet by gut microbial interaction governing cardiovascular disease.

## Development of the human microbiome

Microbiomes develop across body sites in early life, a process that adheres to the principals of ecological succession^33^ and shapes physiological and immunological function^18^. Indeed, bacteria detected at 4 days of age in human neonates correlate with community structure at 120 days of age^34^, indicating the importance of founder organisms in microbiological successional trajectories. Early-life microbiomes therefore may offer insights into the origins of disease and the capacity to both identify those at risk and intervene early to prevent disease development. For example, gut microbiome and metabolic dysfunction in infancy is characteristic of higher-risk for atopy and asthma development in childhood^7^. Moreover, specific microbial-derived metabolites found in elevated concentrations in the feces of high-risk for asthma infants promote key features of immune dysfunction characteristic of established disease^7,35^. For example, the oxylipin 12,13-DiHOME, elevated in the feces of high-risk babies, induces allergic inflammation in both primary immune cell and murine models^35^. Genes encoding bacterial epoxide hydrolases catalyzing the production of 12,13-DiHOME were enriched in the feces of high-risk babies. Introduction of these bacterial genes to the gut microbiome of mice was sufficient to increase circulating concentrations of 12,13-DiHOME and exacerbate airway allergic inflammation^35^. Moreover, in one month infant stool samples increasing concentrations of this oxylipin or the copy number of bacterial epoxide hydrolases capable of its production, significantly increased the risk for atopy and/or asthma development in childhood^35^, underscoring a role for early-life microbes and their metabolic products in disease development. More recently, both direct (electron microscopy) and indirect (molecular) evidence for the presence of a sparse, but viable, bacteria in the human fetal intestine by mid-gestation was reported in humans, and fetal bacterial strains isolated only in the presence of pregnancy hormones exhibited the ability to modulate fetal T-cell inflammatory ability^36^. Independently, development of antigen-experienced immune cell populations with the capacity to respond to microbial stimuli has been demonstrated as early as the second trimester of pregnancy in humans^37–40^ and continues post-natally. Thus vertically, horizontally, and environmentally acquired early-life microbes and their metabolites influence immune function and physiological development in a manner that shapes trajectories with life-long implications for health and an improved understanding of interactions that govern this process is critical.

## Outlook

Ecologically, low-abundance species and strain populations are essential reservoirs of genetic and functional diversity^41^. Broader understanding of primary and ancillary microbial functions amassed during the microbiome assembly process in early life and reassembly following perturbation in mature microbiomes is key to understanding features of microbiome function, stability and resilience. Critical also to this understanding is the role of microbe–microbe and microbe–host interactions which govern competitive colonization and niche specificity^42,43^ in the context of nutritional substrate availability. Immunological immaturity is observed in germ-free and laboratory mice, as compared to wild mice^44–46^ and humans residing on farms exhibit greater microbial functional diversity and a lower susceptibility to chronic inflammatory disease^47,48^. Traditional hunter-gatherer populations possess dynamic lineages of microbes which exhibit seasonal changes and include microbial species largely extinct in urban dwellers^49^, while lifestyle changes including settled habitation lead to depletion of disease protective microbes in nomadic populations^50^. Thus, progressive loss of the most flexible and responsive microbes to environmental exposures may occur at the expense of lifestyle changes and modernization. Microbial ecosystem management, involving precision nutrition and rational microbial supplementation to promote or reinstate microbial functional networks eroded by Western lifestyles and urban exposures may become increasingly important.

As we move into the next decade of human microbiome research, forward momentum in the field requires an understanding of microbial function, productivity, and interaction with the human host across spatial, temporal, and environmental gradients. Integrative analyses of parallel high-resolution cellular profiling approaches applied to longitudinally collected samples capturing microbiome function, productivity, host response and anthropologic measurements will lead to a broader appreciation of our co-evolution with microbes and the forces that shape microbe–microbe–host interactions. In addition to breadth, such studies also require depth; interrogation across scales from the ecosystem level to cellular and molecular networks that shape human biology are necessary to facilitate mechanistic insights necessary to leverage this field for precision diagnostics and interventions. Determining how to successfully re-populate depleted microbial functions and rationally and sustainably re-engineer microbiomes across a range of developmental stages, host genetic backgrounds and environmental exposures represents the next frontier in human microbiome research.
